# Bifractionated CPT-11 with LV5FU2 infusion (FOLFIRI-3) in combination with bevacizumab: clinical outcomes in first-line metastatic colorectal cancers according to plasma angiopoietin-2 levels

**DOI:** 10.1186/1471-2407-13-611

**Published:** 2013-12-27

**Authors:** Stefano Kim, Erion Dobi, Marine Jary, Franck Monnien, Elsa Curtit, Thierry NGuyen, Zaher Lakkis, Bruno Heyd, Serge Fratte, Denis Cléau, Najib Lamfichekh, Virginie Nerich, Boris Guiu, Martin Demarchi, Christophe Borg

**Affiliations:** 1 Department of medical oncology, University Hospital of Besançon, Besançon, France; 2 Department of digestive and liver surgery, University Hospital of Besançon, Besançon, France; 3 Department of gastroenterology, Hospital of Belfort-Montbeliard, Montbeliard, France; 4 Department of gastroenterology, Hospital of Vesoul, Vesoul, France; 5 Department of surgery, Hospital of Belfort-Montbeliard, Montbeliard, France; 6 Department of pharmacy, University Hospital of Besançon, Besançon, France; 7 Department of radiology, University Hospital of Dijon, Dijon, France; 8 INSERM, University of Franche-Comté, Unit 1098, Besançon, France; 9 Medical Oncology Unit, J. Minjoz University Teaching Hospital, Boulevard Alexandre Fleming, Besancon F-25000, France

**Keywords:** Colorectal cancer, Bevacizumab, FOLFIRI3, Irinotecan, Angiopoietin-2

## Abstract

**Background:**

Optimization of chemotherapy effectiveness in metastatic colorectal cancers (mCRC) is a major endpoint to enhance the possibility of curative intent surgery. FOLFIRI3 has shown promising results as second-line chemotherapy for mCRC patients previously exposed to oxaliplatin. The clinical efficacy of FOLFIRI3 was never determined in association with bevacizumab in non-previously treated mCRC patients.

**Methods:**

We conducted a phase II clinical trial to characterize the response rate and toxicity profile of FOLFIRI3-bevacizumab as initial treatment for mCRC. Sixty-one patients enrolled in 3 investigation centers were treated with FOLFIRI3-bevacizumab (median of 10 cycles) followed by a maintenance therapy combining bevacizumab and capecitabine. Levels of plasma angiopoietin-2 (Ang-2) were measured by enzyme-linked immunosorbent assay at baseline.

**Results:**

Overall response rate (ORR) was 66.7% (8% of complete and 58% of partial responses). The disease control rate was 91.7%. After a median time of follow-up of 46.7 months, 56 patients (92%) had progressed or died. The median progression free survival (PFS) was 12.7 months (95% confidence interval (CI) 9.7-15.8 months). The median overall survival (OS) was 24.5 months (95% CI: 10.6-38.3 months). Twenty-one patients underwent curative intent-surgery including 4 patients with disease initially classified as unresectable. Most common grade III-IV toxicities were diarrhea (15%), neutropenia (13%), asthenia (10%), and infections (4%). Hypertension-related medications needed to be increased in 3 patients. In multivariate analysis, surgery of metastases and Ang-2 levels were the only independent prognostic factors for PFS and OS. Indeed, baseline level of Ang-2 above 5 ng/mL was confirmed as an independent prognostic factor for progression free survival (HR = 0.357; 95% CI: 0.168-0.76, p = 0.005) and overall survival (HR = 0.226; 95% CI: 0.098-0.53, p = 0.0002).

**Conclusions:**

As front-line therapy, FOLFIRI-3-bevacizumab is associated with an acceptable toxicity and induced promising objective response rates. However, unfavorable clinical outcomes were observed in patients with high levels of angiopoietin-2.

## Background

Recent advances in surgery of metastases, as well as the widespread use of adjuvant chemotherapy, improved the outcomes of colorectal cancer patients [1-3]. For patients diagnosed with metastatic disease or relapsing after oxaliplatin-based adjuvant therapy, optimization of chemotherapy effectiveness is a major endpoint to enhance the possibility of curative intent surgery. For those patients, CPT-11 (irinotecan)-containing chemotherapies are an available option, particularly when combined with biotherapies.

Irinotecan, a campthotecin analogue, inactivates topoisomerase I via its active metabolite SN38. In first-line mCRC patients, the addition of irinotecan (180 mg/m^2^) to LV5FU2 (FOLFIRI) conferred a significant survival advantage over LV5FU2 alone (leucovorin (LV) 200 mg/m^2^ day 1, 5-fluorouracil (5FU) bolus 400 mg/m^2^ day 1 followed by a 46 hours-5FU continuous infusion 2400 mg/m^2^) [4]. Similarly, the addition of irinotecan to bolus of fluorouracil resulted in longer overall survival compared to fluorouracil alone [5]. The clinical benefit of irinotecan was also demonstrated in patients whose disease had progressed after first-line 5FU [6,7].

Irinotecan, administered alone or in combination with LV5FU2 (FOLFIRI), is also a recommended therapy for second-line metastatic diseases in patients previously exposed to oxaliplatin. In this setting, irinotecan as single agent or in combination with LV5FU2 induces a 4% objective response rate and a progression free survival of 2.5 months [8,9]. These results prompted many investigators to examine new approaches to optimize irinotecan-based chemotherapies.

Bifractionated CPT-11 with LV5FU2 (FOLFIRI3) was identified as an effective therapy in patients previously exposed to oxaliplatin-containing chemotherapies [10-12]. In FOLFIRI3 regimen, 100 mg/m^2^ of irinotecan are delivered as a 60 minutes infusion before and at the end of LV5FU2. In a phase II trial, FOLFIRI3 regimen was assessed in patients previously treated with FOLFOX and achieved an objective response rate of 23%. Furthermore, a stable disease was observed in 37% of the patients and the progression free survival was 4.2 months [10]. Bidard FC et al. analyzed the clinical interest of several irinotecan-based protocols delivered in the second-line setting for patients previously included in the OPTIMOX 1 study. These patients were treated by FOLFOX in the first-line setting and were prospectively registered for subsequent treatments. FOLFIRI3 chemotherapy was associated with a significantly better objective tumor response rate (17% compared to 8% with FOLFIRI) and an enhanced progression free survival (3.7 months compared to 3 months with FOLFIRI) [11]. These results suggested that bi-fractionated irinotecan might be a relevant option for patients previously treated with oxaliplatin.

Then, this phase II clinical trial was designed to assess the clinical efficacy and toxicity of FOLFIRI3 in combination with bevacizumab (FOLFIRI3-b), a Vascular Endothelial Growth Factor-neutralizing monoclonal antibody indicated for the first-line treatment of metastatic CRC patients [13].

Although there are currently no biomarkers with proven clinical interest to identify patients eligible to bevacizumab and chemotherapy, a pilot study performed on 34 metastatic colorectal cancer patients treated with chemotherapy and bevacizumab showed that high levels of plasma angiopoietin-2 predict a poor prognosis [14]. Therefore, we decided to perform an ancillary study to further substantiate the prognostic value of baseline angiopoietin-2 plasma levels in patients treated with FOLFIRI3 and bevacizumab.

## Methods

### Study design

The main objective of this single-arm phase II study was to determine the objective response rate of FOLFIRI3-b for patients undergoing first-line treatment of metastatic colorectal cancer. Secondary objectives were the monitoring of side effects, the assessment of median progression free and overall survival and the characterization of angiopoietin-2 prognostic value. Sixty-one patients were enrolled in two general hospitals and a university hospital. The study was conducted in accordance with the Declaration of Helsinki, the International Conference on Harmonization Guidelines for Good Clinical Practice. Each patient gave written consent before entering the study. The protocol was approved by the ethical committee CPP Est-II and registered on ClinicalTrials.gov (study NCT00544011).

### Eligibility

Patients with first-line mCRC and measurable lesions were included. Previous adjuvant chemotherapy was allowed provided that the last administration was given at least 6 months before randomization. Other eligibility criteria were: age 18 to 80 years, Eastern Cooperative Oncology Group Performance Status 0, 1 or 2 and written informed consent. Exclusion criteria included: previous bevacizumab treatment; proteinuria exceeding 1 g/24 h; coagulopathy; inadequate hematologic function (absolute neutrophil count < 1.0 × 10^9^/L, platelets < 100 × 10^9^/L); inadequate hepatic function (total bilirubin > 1.5× upper limit of normal (ULN), serum transaminases > 2× ULN in absence of liver metastases or > 5× ULN in presence of liver metastases); inadequate renal function (clearance of creatinine < 30 ml/min); major surgery <28 days before inclusion; non-controlled cardiovascular disease; non-controlled hypertension; active hemorrhagic event; aspirin more than 325 mg/day treatment. As the main objective was to assess the tumor response rate, patients amenable to curative intent surgery were included in the study. Surgery of metastases was allowed after 6 cycles of FOLFIRI3-b and the precise timing left at the discretion of investigators.

### Treatment

FOLFIRI3-bevacizumab regimen consisted of bevacizumab 5 mg/kg day 1, irinotecan 100 mg/m^2^ day 1, LV 400 mg/m^2^ day 1 followed by a 46-h 5-FU continuous infusion (2400 mg/m^2^), and irinotecan 100 mg/m^2^ at day 3 [12]. Induction treatment was administrated every 2 weeks for a maximum of 12 cycles, until disease progression, unacceptable toxicities, surgical intervention, or withdrawal of consent.

Maintenance treatment consisted of bevacizumab 7.5 mg/kg intravenous infusion over 60 minutes, and capecitabine 1000 mg/m^2^ day 1 to 14, every 3 weeks until tumor progression, unacceptable toxicity, or withdrawal of consent.

Dose reductions were required for all grade 3 or 4 toxicities attributed to study medications. Bevacizumab was not dose reduced but delayed or discontinued in patients with grade 3 or 4 toxicities.

### Safety and efficacy assessment

At baseline and before each cycle, clinical and biological examinations were performed. Adverse events were evaluated continuously and graded according to National Cancer Institute Common Toxicity Criteria (NCI CTC) version 3. The response was defined according to Response Evaluation Criteria In Solid Tumors (RECIST) v1.0. Radiological response assessment was validated in a blinded fashion by an independent radiologist. Tumor responses were assessed every eight weeks by spiral computed tomography. Resectability of metastases was determined by IRFC-multidisciplinary gastrointestinal oncology team before treatment initiation and when the best response was achieved.

### Plasma sample collection and analysis

Blood samples were drawn at baseline and immediately processed for plasma and serum freezing at -80°C. Samples frozen more than 4 hours following venous blood collection were not included in the analysis. Enzyme-linked immunosorbent assays (ELISA) were used to measure angiopoietin-2 in serum samples and VEGF-A in plasma samples (RnD systems) according to the manufacturer’s instructions. Each sample was analyzed in duplicate.

### Statistical analysis

The primary endpoint of the trial was to determine the objective response rate to the FOLFIRI3 sans espace bevacizumab regimen in patients with first-line mCRC cancers. The study was designed as an exploratory, pilot, single-arm, multicenter, phase II study. A Simon two-stage (optimal) design was used. At least 11 out of the first 21 patients needed to achieve an objective response in stage 1 for recruitment to continue until a target of 45 fully evaluable patients had been reached. Based on standards available at the time of study design, we considered the standard objective response rate (H0 hypothesis) achieved with FOLFIRI and bevacizumab as 50%. Based on an α and β error probabilities of 0.1 respectively, we considered that if an objective response (complete and partial response) was observed in at least 26 out of 45 patients with metastatic colorectal cancers, the regimen would merit further evaluation in prospective subsequent trials. Moreover, regarding the high number of patients who underwent a curative intent surgery, the cohort was extended to 61 patients to include a total of 40 patients with non-resectable disease. Survival data were computed according to Kaplan-Meier method and analyses were performed on an intent-to-treat population including all registered patients. Secondary endpoints were PFS, OS, toxicity profile, metastases resection rate, and biologic analysis of possible predictive factors of efficacy and toxicity. PFS was defined as the time from study enrolment to the first documentation of progressive disease (PD) or death from any cause. Overall survival was defined as the time from study enrolment to death from any cause.

## Results

### Baseline characteristics

Sixty-one patients were enrolled in the study between October 2007 and July 2009. The median age of the patients was 63.6 years (range, 38–81). Fifty-seven patients (93%) had an Eastern Cooperative Oncology Group Performance Status (ECOG PS) 0–1. Thirty-five patients (57%) presented only one metastatic site and 7 patients (12%) had 3 or more visceral sites involved by metastases. Twenty-five patients (41%) had liver-limited metastases. Twenty-two patients (36%) displayed metachronous metastases at inclusion. Eighteen patients (30%) had received prior adjuvant chemotherapy, including an oxaliplatin-based chemotherapy for 15 patients (25% of the whole cohort). Primary tumor was resected in 3 out of the 39 synchronous metastatic patients. Two primary tumor resections were performed before enrolment due to a symptomatic disease and one resection was performed after enrolment due to an occlusion (Table 1).

**Table 1:** Patient’s characteristics

	**FOLFIRI3-bevacizumab**	
	**(n = 61)**	
**Demographic or clinical characteristics**	**No. of patients**	**%**
Male	35	57
Age, years		
Median	63.6	
Range	38-81	
ECOG performance status		
0	25	41
1	32	52
2	4	7
Primary tumor type		
Colon	38	62
Rectal	23	38
No. of sites involved by metastases		
1	35	57
2	19	31
> = 3	7	12
Site of metastatic disease		
Liver only	25	41
Liver + other	20	33
Other only	16	26
Prior adjuvant chemotherapy		
All	18	30
Oxaliplatin-based	15	25
< 12 months	8	13
Synchronous tumors		
Yes	39	64
No	22	36

### Administration of FOLFIRI3-bevacizumab

All patients accomplished at least one cycle of treatment. Patients received a median of 10 cycles of induction chemotherapy (range 2–14) and 40 patients completed all planned cycles of FOLFIRI3-b. Twenty-one patients (34%) discontinued their induction treatment: 6 for progression, 3 for treatment-related toxicities and 12 for programmed metastasis surgery. The relative dose intensity (calculated from FOLFIRI3-b initiation to the last course of chemotherapy delivered before toxicity, disease progression, surgery, or the planned 12^th^ cycle of FOLFIRI3-b) was 88% for Irinotecan, 85% for 5-FU, and 87% for bevacizumab. Median dose administered for each cycle was 91% for irinotecan, 95% for 5-FU, and 100% for bevacizumab.

### Safety

Toxicity analysis was performed in the intent-to-treat population (61 patients, Table 2). Twenty patients (33%) suffered ≥ grade III treatment-related side effects. One patient (1.6%) died during induction regimen due to treatment-related toxicity (colitis and sepsis). Moreover, one patient with synchronous metastases exhibited a colon cancer bleeding requiring surgery. Post-operative recovery was achieved within 7 days and chemotherapy was reintroduced at day 28 post-surgery. Most common grade III-IV toxicities were diarrhea (15%), neutropenia (13%), asthenia (10%), and infections (4%). Hypertension-related medications needed to be increased in 3 patients.

**Table 2:** Grade 3 and 4 adverse events: NCI CTC v.3

	**Induction chemotherapy**		**Maintenance chemotherapy**	
	**(N = 61)**		**(N = 41)**	
**Adverse events**	**No. of patients**	**%**	**No. of patients**	**%**
Nausea	1	2	0	-
Vomiting	1	2	0	-
Diarrhea	9	15	2	5
Mucositis	0	-	0	-
Fatigue	6	10	1	2
Platelet count decreased	0	-	0	-
Anemia	0	-	0	-
White blood cell decreased	3	5	0	-
Neutropenia	8	13	0	-
	(2 grade IV)			
Febrile neutropenia	1	2	0	-
Hypertension	3	5	1	2
Hemorrhage	1	2	0	-
Thromboembolic event	0	-	1*	2
Palmar-plantar syndrome	0	-	6	15

*One catheter related deep vein thrombosis.

Grade IV toxicity: the only grade IV toxicity observed was neutropenia.

### Efficacy

Among the 60 assessable patients, ORR was 66.7% (95% CI: 55–79) including 58.4% of partial responses and 8.3% of complete responses (CR). Stable disease (SD) was observed in 25% of patients conferring to this strategy a disease control rate of 91.7%. An independent review of the radiological responses was performed and showed only one difference: one patient was considered to be in CR by our investigators and in non-measurable SD by the independent committee. However, later liver resection confirmed histological CR.

After a median time of follow-up of 46.7 months (data base up-dated in December 2012), 56 patients (92%) had progressed. The median PFS was 12.72 months (95% CI: 9–16 months). So far, 42 patients (68.8%) have died and median OS was 24.5 months (95% CI: 10.6-38.3 months). Of note, none of the patient’s characteristics influenced the clinical results achieved with FOLFIRI3-bevacizumab treatment. Particularly, the ORR was 58.3% in patients above 70 year-old compared to 68.8% for patients less than 70 year old at the inclusion (p = 0.51). Moreover, FOLFIRI3-bevacizumab was also effective in patients treated with oxaliplatin-based adjuvant chemotherapy (ORR of 60%, p = 0.54).

### Surgery of metastases

**Figure 1:**
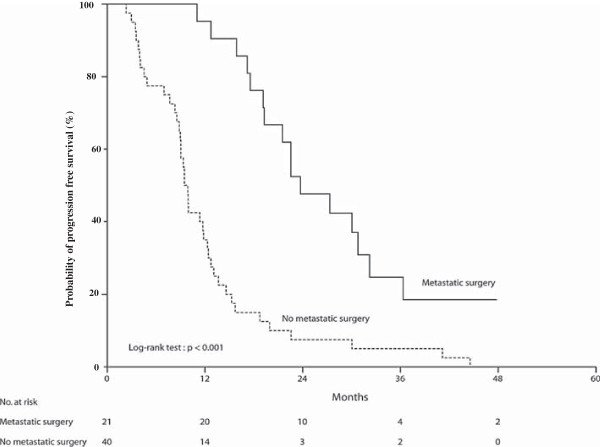
**Kaplan Meier curve for progression free survival of metastatic colorectal cancer patients treated by FOLFIRI3-bevacizumab in the first line setting.** The probability of progression free survival is reported in the 21 patients treated with FOLFIRI3-bevacizumab who underwent a surgery of their metastases and in the 40 patients with unresectable disease.

Twenty-one patients (34%) underwent curative-intent surgery of their metastases. A complete histological response was observed in 5 patients. R0 resection was achieved in twenty patients, while the resection status was considered as R1 for one patient. Four of these patients were initially considered as non-resectable, including one patient with 2 sites involved by metastases (lung and liver). Five patients (24%) among the 21 patients who have undergone metastasis surgery were still in complete remission at the time of the last analysis. The median progression free survival was 23.6 months (95% CI: 17.3-29.9 months) in patients who underwent a surgery of their metastases compared to 9.4 months (95% CI: 8.47-10.33 months) for patients with non-resected metastases (Figure 1; p < 0.001). The median overall survival was not reached in patients who underwent a metastatic surgery, while median OS was 18.33 months (95% CI: 15.15-21.5 months) in patients with unresectable disease (p < 0.001).

### Feasibility and efficacy of maintenance treatment by capecitabine-bevacizumab

Twenty patients did not receive maintenance chemotherapy: 1 patient died during induction regimen, 11 patients displayed progressive diseases before capecitabine-bevacizumab initiation, 8 patients underwent curative-intent surgery and no further treatment was proposed. Then, capecitabine-bevacizumab therapy was administered to 40 eligible patients and one more patient, who refused capecitabine, received only bevacizumab.

A total of 346 cycles and a median of 8.7 cycles was administered (range 1–33) per patient at the time of analysis. Median dose of capecitabine *per* cycle and *per* patient was 83.4%.

The treatment was generally well tolerated. No grade IV toxicity was recorded and 20% of patients experienced grade III toxicities. Most frequent grade III toxicities were hand-foot syndrome (15%), and diarrhea (5%). Bevacizumab had to be stopped before capecitabine in only one case due to catheter-related deep vein thrombosis. Overall, maintenance treatment was discontinued for toxicity or withdrawal of consent in 2 patients (5%). The median duration of disease control from first cycle of maintenance therapy was 8 months (range, 1–38).

### High levels of plasma angiopoietin-2 at baseline correlate with poor clinical outcomes in patients treated with FOLFIRI3-b

**Figure 2:**
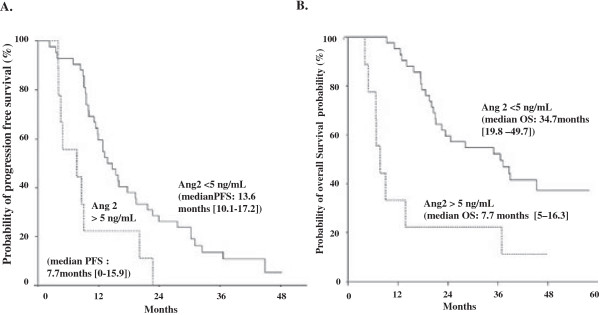
**Kaplan Meier curves for progression free survival and overall survival of metastatic colorectal cancer patients treated by FOLFIRI3-bevacizumab in the first line setting, according to the baseline level of angiopoietin-2. (A)** The probability of progression free survival was reported in patients with baseline angiopoietin-2 levels below 5 ng/mL or above 5 ng/mL. **(B)** The probability of overall survival is shown according to baseline angiopoietin-2 levels. Patients with baseline angiopoietin-2 levels above 5 ng/mL displayed a significantly decreased OS (p = 0.0002).

In 51 patients, plasma and serum were available at baseline for analysis. Among angiogenic factors, angiopoietin-2 (Ang-2) was recently proposed in a cohort of 34 patients, as a candidate biomarker for outcomes of mCRC patients treated with bevacizumab-containing chemotherapy [14]. Then, we decided to monitor Ang-2 and VEGF-A levels in this study. Seven patients included in this phase II clinical trial had increased VEGF-A levels compared to normal volunteers or stage II-III colorectal cancers. However, we did not observe a significant negative influence of increased VEGF-A levels on the ORR (83%), PFS (10.7 months) or OS (20.6 months). A preliminary set of experiments confirmed that Ang-2 levels remain below 5 ng/mL in all normal volunteers (n = 20) or stage II-III colorectal cancers (n = 20), in line with the results of Goede et al. [14]. Ang-2 plasma levels above 5 ng/mL at baseline were observed in nine patients (17.3%) of our cohort. ORR was 44% in patients with increased levels of Ang-2, compared to 74.4% in patients with Ang-2 levels below 5 ng/mL. The median PFS was 7.7 months (95% CI: 0–15.9 months) in patients with high Ang-2 levels compared with 13.6 months (95% CI: 10.1-17.2 months) in patients with Ang-2 levels below 5 ng/mL (Figure 2A). Furthermore, overall survival was significantly better in patients with low levels of Ang-2 (median OS: 34.7 months; 95% CI: 19.8-49.7) than in patients with high levels of Ang-2 (median OS: 7.7 months; 95% CI: 5–16.3 months; Figure 2B). In multivariate analysis, surgery of metastases and Ang-2 levels were the only independent prognostic factors for PFS and OS. Indeed, a level of Ang-2 above 5 ng/mL was confirmed as an independent prognostic factor for progression free survival (HR = 0.357; 95% CI: 0.168-0.76, p = 0.005) and overall survival (HR = 0.226; 95% CI: 0.098-0.53, p = 0.0002). Altogether, these results confirm the clinical interest of angiopoietin-2 monitoring to predict PFS and OS in mCRC patients and suggest a decreased efficacy of FOLFIRI3-b in these patients.

## Discussion

Irinotecan inhibits DNA replication by interfering with topoisomerase I. Topoisomerase enzymes display a helicase activity involved in DNA repair. Then, it might be possible that topoisomerase blockage following DNA damage could prevent DNA repair and enhance apoptosis induced by chemotherapy. Indeed, preclinical evidence suggested that the anti-proliferative activity of 5FU and irinotecan combination is schedule dependent [15-18]. In line with the above hypothesis, several studies showed that a delayed administration of irinotecan increases FOLFIRI cytotoxicity. FOLFIRI2 regimen (irinotecan delivery at the end of a modified LV5FU2 chemotherapy) in heavily pretreated CRC patients induced promising objective responses but also a limiting hematologic toxicity [19]. As reported here and in previous studies, FOLFIRI3 has a more appropriate toxicity profile [12]. In addition, bifractionated irinotecan combined to LV5FU2 (FOLFIRI3) was shown to be active in mCRC resistant to FOLFIRI and has been correlated to a longer PFS in second line treatment of mCRC patients [10-12]. However, the clinical interest of this regimen was never investigated in the first-line setting, where the best chemotherapy to combine with bevacizumab is still a matter of debate.

In a pivotal randomized clinical trial, bevacizumab combination with IFL (weekly irinotecan plus bolus 5-fluorouracile/leucovorin) improved both median PFS and median OS in previously untreated mCRC patients. Patients receiving both bevacizumab and IFL had a median PFS of 10.6 months and a median OS of 20.3 months, while median PFS and OS were 6.2 and 15.6 months respectively for patients treated with IFL alone [13]. FOLFIRI and bevacizumab efficacy was primarily assessed in retrospective studies. López R et al. reported a 50.5% objective response rate, a 10.6 month median PFS and a 20.7 month median OS in first line treatment of mCRC patients [20]. Seventy one percent of these patients had liver metastases and 14% of them underwent curative-intent surgery. In this study, adverse events were the cause of treatment discontinuation for 8% of the patients compared to 5% in our study. In a recently reported phase II trial including 43 patients, FOLFIRI and bevacizumab achieved a 65% response rate, a 12.8 months median PFS and a median OS of 31.9 months [21]. Adverse events were the cause of treatment discontinuation in 8 patients and curative-intent surgery was performed in 4 patients [21]. Of note, the addition of bevacizumab to FOLFOX has shown similar results with a response rate of 52% and a median PFS of 9.9 months [22].

The BICC-C study contributed to clarify the relative interest of three different irinotecan-based chemotherapies in the first line treatment of mCRC. The addition of oral capecitabine to irinotecan (CapeIRI) induced an enhanced level of toxicities. Furthermore, median overall survival was significantly higher for patients treated with FOLFIRI-bevacizumab (median OS of 28 months vs 19.2 months for mIFL-treated patients) [23].

In the current study, we found that FOLFIRI3- bevacizumab was a manageable option in first line metastatic colorectal cancer patients. Indeed, side effects and toxic deaths observed in our study are comparable to those reported by other clinical trials. The ratio of adverse events leading to death was 1.6% in our study compared to 2.6% and 2% in the pivotal study reported by Hurwitz et al. and by the BEAT study respectively [13,24].

Moreover, the present trial confirms the feasibility of a maintenance chemotherapy combining capecitabine and bevacizumab following FOLFIRI3-b. Maintenance chemotherapy might be a convenient fashion to extent the period of disease control while minimizing the cumulative side effects [25-28]. Our results indicate that bevacizumab and capecitabine maintenance chemotherapy is feasible after FOLFIRI3-bevacizumab, since no grade IV toxicity occurred and only two patients out of 41 exposed to this strategy, discontinued capecitabine following side effects.

A second conclusion that can be drawn from the current study is that survival achieved in patients treated by FOLFIRI3-b is similar to those reported by previous clinical trials (Table 3). The median PFS and OS observed in patients with unresectable metastases were 9.4 months (95% CI: 8.47-10.33 months) and 18.33 months (95% CI: 15.15-21.5 months) respectively. Of note, patients included in the CONcePT trial achieved a median PFS of 12 months [27], while patients included in the MACRO trial and treated with Xelox-bevacizumab followed by bevacizumab alone as a maintenance therapy displayed a median PFS of 9.7 months and a median OS of 20 months [28]. Moreover, we cannot exclude a negative impact of FOLFIRI3-b regimen on patient’s quality of life. Indeed FOLFIRI3-b requires the administration of irinotecan twice per cycle (before and at the end of continuous 5FU), extending both length of hospitalization and healthcare travel costs.

**Table 3:** Review of the main clinical trials performed in first line metastatic colorectal cancer

	**Chemotherapy**	**Liver metastases only (%)**	**One metastatic site only (%)**	**ORR (%)**	**mPFS (months)**	**mOS (months)**	**Ref**	**Comments**
**Tournigand C et al. **[9]	FOLFIRI	NR	56	56	8.5	21.5	J Clin Oncol 2004; 22(2):229–37.	
**Colluci G et al. **[34]	FOLFIRI	50	56	31	7	14	J Clin Oncol 2005; 23:4866–4875.	
**Labiancha R et al. **[35]	FOLFIRI	NR	NR	42	6	18	Annals of Oncology 2011; 22:1236–1242.	
**Koopman M (CAIRO study) **[36]	FOLFOX or FOLFIRI	23	NR	41	7,8	17.4	Lancet 2007; 370:135–42.	
**Ducreux M (FFCD2000-05) **[37]	FOLFOX	NR	52	58	7.6	16.2	Lancet Oncol 2011; 12:1032–44.	
**Chibaudel B (OPTIMOX2 study) **[25]	FOLFOX7	NR	42	59.6	8,6	23.8	J Clin Oncol 2009; 27:5727–5733.	Maintenance arm
**Maughan TS (COIN study arm 1) **[38]	FOLFOX	21	35	57	8,6	17.9	Lancet 2011; 377(9783):2103–2114.	
**Hurwitz H et al. **[13]	IFL + Bev	NR	37	44.8	10.6	20.3	N Engl J Med 2004; 350:2335–42.	
**Fuchs CS (BICC study) **[23]	Folfiri-bev	NR	NR	57.9	11.2	28	J Clin Oncol 2007; 25:4779–4786.	
**SALTZ LB (NO16966) **[39]	FOLFOX + bev	NR	43		9.4	21.3	J Clin Oncol. 2008; 26(12):2013–9.	
**Schmoll HJ (Horizon study) **[40]	FOLFOX + bev	22	45	47	10.3	22.3	J Clin Oncol 2012; 30:3588–3595.	
**Van Cutsem(Crystal study) **[41]	FOLFIRI cetux	21,5	NR	57,2	9.9	23.5	J Clin Oncol 2011; 29:2011–2019.	Kras wt only
**Maughan TS (COIN study arm 2) **[38]	FOLFOX cetux	24	37	64	8.6	17	Lancet. 2011; 377(9783):2103–2114.	Kras wt only
**Douillard JY (Prime study) **[42]	FOLFOX pamitumumab	18	21	55	9.6	23.9	J Clin Oncol 2010; 28:4697–4705.	Kras wt only
**Bokemeyer (OPUS study) **[43]	FOLFOX + cetux	30	44	58	8.3	22.8	Annals of Oncology 2011; 22:1535–1546.	Kras wt only
**Masi G **[44]	FOLFOXIRI	34	55	60	9.8	23.4	J Natl Cancer Inst 2011;103:21–30.	
**Masi G **[30]	FOLFOXIRI beva	53	58	77	13.1	30.9	Lancet Oncol 2010; 11:845–52.	

(NR: not reported).

Nevertheless, a possible clinical interest of FOLFIRI3-b might be the high level of tumor shrinkage observed in our study. Indeed, the 66.7% ORR observed in the FOLFIRI3-bevacizumab study may be considered for patients with primary unresectable disease in whom downsizing is mandatory to allow metastasis surgery. The correlation established between the response rate and the resection rate supports chemotherapy optimization in mCRC patients who might potentially become candidate for surgery [29]. The high ORR observed in our study might reflect a favorable selection of patients as suggested by the ratio of patients with only one site involved by metastases (57%). A review indicating the ORR, mPFS and mOS achieved in recent clinical trials according to the ratio of patients with only one metastatic site is summarized in Table 3. FOLFIRI3 and bevacizumab combination compares favorably with other regimen.

Promising results have also recently been reported in 57 mCRC patients treated with FOLFOXIRI and bevacizumab. 44 of these patients (77%) achieved a partial response and 26% of them underwent a radical surgery of their metastases. FOLFOXIRI-bevacizumab led to a median PFS and an OS of 13.1 and 30.9 months [30]. Then, FOLFIRI3-bevacizumab might be of particular interest in patients previously treated with oxaliplatin-containing adjuvant chemotherapy and not eligible to FOLFOX or FOLFOXIRI regimen, especially if the induction of a tumor shrinkage is require to allow optimal resection of the metastatic disease. Of note, an ORR of 60% was achieved after FOLFIRI3-b treatment in patients exposed to oxaliplatin.

Finally, our study confirmed the prognostic value of plasma angiopoietin-2 levels in metastatic CRC. This result is of particular interest since most angiogenic related biomarkers might be related to prognosis only when monitored after treatment initiation. Here, a baseline Ang-2 level above 5 ng/mL was confirmed as an independent prognostic factor for progression free survival (HR = 0.357; 95% CI: 0.168-0.76, p = 0.005) and overall survival (HR = 0.226; 95% CI: 0.098-0.53, p = 0.0002).

To our knowledge, angiopoietin-2 monitoring was not reported in randomized clinical trials, precluding any conclusion regarding a potential predictive value of this biomarker.

However, our results are in agreement with the previous report of Goede et al., where high levels of angiopoietin-2 had a prognosis value and correlated with a decreased survival in a cohort of 34 colorectal cancer patients treated by chemotherapy and bevacizumab [14].

Several evidences support the direct role of angiopoietin-2 in cancer prognosis. First, angiopoietin-2 expression was shown to be correlated with colorectal cancer stages and progression [31]. In addition, angiopoietin-2 was identified as an independent prognosis biomarker in myeloid leukemia and melanoma patients not treated with anti-angiogenic therapies [32,33].

## Conclusion

In conclusion, front-line therapy with FOLFIRI-3-bevacizumab is associated with an acceptable toxicity. A promising objective response rate was achieved, particularly in patients previously exposed to oxaliplatin. However, unfavorable clinical outcomes were observed in patients with high levels of angiopoietin-2.

## Competing interests

The authors declare that they have no competing interests.

## Authors’ contributions

SK, ED, MJ, TNG, ZL, BH, SF, DC; NL, MD and CB included patients; SK, EC, CB wrote the manuscript; FM and VN performed statistical analysis; BG performed RECIST criteria assessment; MJ, ED performed Angiopoietin monitoring, SK and CB analyzed results. All authors read and approved the final manuscript.

## Pre-publication history

The pre-publication history for this paper can be accessed here:

http://www.biomedcentral.com/1471-2407/13/611/prepub
